# Unique Cytokine Response in West Nile Virus Patients Who Developed Chronic Kidney Disease: A Prospective Cohort Study

**DOI:** 10.3390/v13020311

**Published:** 2021-02-17

**Authors:** Michael Hansen, Melissa S. Nolan, Rodion Gorchakov, Rodrigo Hasbun, Kristy O. Murray, Shannon E. Ronca

**Affiliations:** 1 Department of Family and Community Medicine, Baylor College of Medicine, Houston, TX 77030, USA; Michael.Hansen@bcm.edu; 2 Department of Epidemiology and Biostatistics, Arnold School of Public Health, Columbia, SC 29208, USA; MSNOLAN@mailbox.sc.edu; 3 Department of Pediatrics, Section Tropical Medicine, Baylor College of Medicine, Houston, TX 77030, USA; rodion.gorchakov@gmail.com (R.G.); kmurray@bcm.edu (K.O.M.); 4 Department of Internal Medicine, The University of Texas Health Science Center at Houston, Houston, TX 77030, USA; Rodrigo.Hasbun@uth.tmc.edu

**Keywords:** West Nile virus, chronic kidney disease, cytokine, chemokine, proinflammatory, *Flavivirus*

## Abstract

West Nile virus (WNV) is a widespread and devastating disease, especially in those who develop neuroinvasive disease. A growing body of evidence describes sequelae years after infection, including neurological complications and chronic kidney disease (CKD). Eighty-nine out of 373 WNV-positive cases were followed for approximately two years and compared to 127 WNV-negative controls with and without CKD. Adjusted risk ratios (aRRs) were calculated via a log binomial regression to determine the impact of WNV exposure and other possible confounders on the likelihood of developing CKD. Cytokine profiles of WNV patients and controls were evaluated to characterize differences and describe potential underlying pathophysiological mechanisms. The associated risk for developing CKD was significantly associated with history of WNV infection (aRR = 1.91, 95% CI 1.13–3.25). Additionally, five distinct cytokines were found to be significantly associated with WNV infection (eotaxin, IL-8, IL-12p70, IP-10, and TNFα) after the *p*-value was adjusted to <0.0019 due to the Bonferroni correction. These data support that WNV infection is an independent risk factor for CKD, even after accounting for confounding comorbidities. WNV participants who developed CKD had high activity of proinflammatory markers, indicating underlying inflammatory disease. This study provides new insights into CKD resultant of WNV infection.

## 1. Introduction

West Nile virus (WNV) is a *flavivirus* of great public health importance in the United States. National estimates indicate that nearly 7 million persons have been infected [1]. Furthermore, a 2014 study by Staples et al. determined that the total cost of hospitalized WNV infections from 1999 to 2012 exceeded $778 million [2]. While approximately 80% of WNV infections are asymptomatic, the 20% of symptomatic infections manifest in two broad categories: West Nile fever (WNF) and West Nile neuroinvasive disease (WNND). WNND is further divided into encephalitis (WNE), meningitis (WNM), and acute flaccid paralysis (AFP), which account for ~1% of infections. Mild symptomatic infections are often undiagnosed or misdiagnosed due to nonspecific presentation and paucity of testing [3].

It is well established that infection with WNV can lead to severe morbidity, especially in WNND patients, including neurocognitive impairment, debilitating fatigue, persistent neuromuscular paralysis, tremors, depression, anxiety, regional brain atrophy, and possibly kidney damage [4,5,6,7]. These long-term conditions have been reported to effect greater than 40% of study participants up to eight years after initial infection [5]. In addition to severe, long-term disabilities, we have identified premature death as a result of renal and infectious causes [8] and have evidence that WNV is shed in the urine of a subset of cases as much as nine years after infection, with WNV visualized in the kidney and urine of patients by electron microscopy [9]. In a prospective cohort study by Nolan et al., WNV patients experience chronic kidney disease (CKD) more often than one would anticipate, with up to 40% of WNV-positive individuals diagnosed with CKD up to 9 years after WNV disease. WNND was found to be an independent risk factor for the development of all stages of CKD, even when controlling for hypertension and diabetes [7]. This growing body of evidence leads to significant concerns for the development of chronic renal disease as a long-term consequence of WNV infection. By comparing a WNV patient cohort to unexposed controls, we further analyzed the correlation between WNV and CKD. We also sought to identify potential cytokines that may be uniquely associated with kidney disease in WNV patients.

## 2. Materials and Methods

### 2.1. Patient Population

Since 2002, a cohort of WNV patients in Houston, Texas was maintained and followed prospectively from the time of enrollment. This study received human subject’s ethics approval from the Institutional Review Boards at the University of Texas Health Sciences Center at Houston in 2002 and then Baylor College of Medicine under H30533. Participants gave written informed consent prior to enrollment and agreed that their specimens could be used for future testing purposes. To date, this cohort consists of 373 enrolled participants with a confirmed history of WNV infection. Of the participants, 89 WNV cases agreed to give additional samples at least 3 months apart for the purpose of this study to verify glomerular filtration rates (GFRs). All 89 participants were beyond the acute phase of infection. At the time of enrollment, the shortest interval between resolution of WNV symptoms and sample collection was 6 months, and the longest interval was 10 years. Controls were recruited from a Houston-based family medicine clinic and were confirmed to be WNV serologically negative. Of the 127 controls, a subset were randomly selected (*n* = 12) for cytokine testing.

Complete metabolic profiles and blood counts were performed on blood samples and sent to Quest Diagnostic Laboratories, Inc. Results from these tests were aggregated using the Modified Diet in Renal Disease (MDRD) formula to determine the calculated glomerular filtration rate (GFR), as is recommended over the CKD-EPI measure by the National Institute of Diabetes and Digestive and Kidney Diseases when estimated GFR (eGFR) rates are expected to be less than 60 milliliters per minute (mL/min) [10]. Chronic kidney disease was defined according to Kidney Disease Outcomes Quality Initiative (KDOQI) criteria for stages 3 through 5 (eGFR < 60) sustained for 3 months or more [11].

### 2.2. Cytokine Panel

Literature review of the most common cytokines associated with arboviral diseases and cellular mechanisms associated with acute and chronic renal injury were assessed and are summarized in Supplemental Table S1 [12,13,14,15,16,17,18,19,20,21,22,23,24,25,26,27,28,29]. Blood samples were on average 4.3 years (std. dev. = 2.7) after acute West Nile virus diagnosis. While several blood samples were used to assess CKD status, only the first sample was used to determine cytokine values. A cytokine panel composed of 26 antiviral and/or pro-inflammatory cytokines and growth factors was performed using a Luminex^®^ IS 100 platform (Austin, TX, USA) with Milliplex Analyst software, and we used an external control to confirm the consistency between panels and plates prior to analysis. The cytokine panel consisted of eotaxin, granulocyte-colony stimulating factor (G-CSF), granulocyte-macrophage colony stimulating factor (GM-CSF), interferon alpha (INF-α), interferon alpha 2 (INF-α2), interleukin 1 alpha (IL-1α), 1 beta (L-I1β), interleukin 2 (IL-2), interleukin 3 (IL-3), interleukin 4 (IL-4), interleukin 5 (IL-5), interleukin 6 (IL-6), interleukin 7 (IL-7), interleukin 8 (IL-8), interleukin 10 (IL-10), interleukin 12p40 (IL-12p40), interleukin 12p70 (IL-12p70), interleukin 13 (IL-13), interleukin 15 (IL-15), interleukin 17 alpha (IL-17α), interferon gamma-induced protein 10 (IP-10), monocyte chemoattractant protein-1 (MCP1), macrophage inflammatory protein 1 alpha (MIP1α), macrophage inflammatory protein 1 beta (MIP1β), tumor necrosis factor alpha (TNFα), and tumor necrosis factor beta (TNFβ).

### 2.3. Analysis

Demographics between WNV-positive and control groups were directly compared using Student’s *t*-test and chi-square analyses. CKD was evaluated using a logistic regression model, however, due to CKD being relatively high in both groups, risk ratios were calculated using a log binomial regression model. Each risk factor was assessed in a univariate model and any risk factors that had a *p*-value less than 0.2 were included in the final adjusted model via a backward stepwise inclusion mechanism that allowed a maximum of six distinct risk factors. Of note, while the initial study design was intended for a matched analysis, recent literature indicates that an unconditional logistic regression model is appropriate—even potentially recommended—in studies utilizing loose matching of demographic features [30]. The final results from the adjusted model were reported with their adjusted risk ratios (aRRs) and corresponding 95% confidence intervals (95% CIs). 

In the cytokine analysis, values were found to be non-normally distributed using the Shapiro–Wilks test. Additionally, variance between each group was found to be un-equal using the Fligner–Killeen test and was mediated by removing outliers that were greater than two standard deviations above the average. The impact of age on cytokine levels was assessed independently and was found to have no significant correlation with any cytokine in our patient population. The multiple groups were then compared using the Kruskal–Wallis H-test followed by a post hoc intergroup comparison using Dunn’s test. Statistical significance was adjusted for multiple comparisons using a Bonferroni correction that lowered the upper-value threshold of the *p*-value from 0.05 to 0.0019. All statistical analyses were performed using open-source R statistical software version 3.6.1 (R-Studio, Boston, MA, USA) [31]. 

## 3. Results

### 3.1. Patient Population

Both WNV-positive cases and controls had similar demographics, with age, sex, and race/ethnicity comparable between each group. Comorbidities were largely the same in both populations, except for a history of heart disease that was significantly higher in the WNV cohort (Table 1). CKD prevalence was found to be approximately 12.6% in the control group and 23.6% in the WNV cohort. The results from the log binomial regression model showed that the adjusted risk ratios were statistically significant for three variables: hypertension (RR = 3.13, 95% CI 1.26–7.77), individuals older than 65 years of age (RR 2.46, 95% CI 1.18–5.09), and a serologic positive history for WNV infection (RR 1.91, 95% CI 1.13–3.25). Diabetes, obesity, and history of heart disease were all found to have no statistically significant association with the development of CKD after adjustment in our population (Table 2).

### 3.2. Cytokine Profiles 

**Figure 1:**
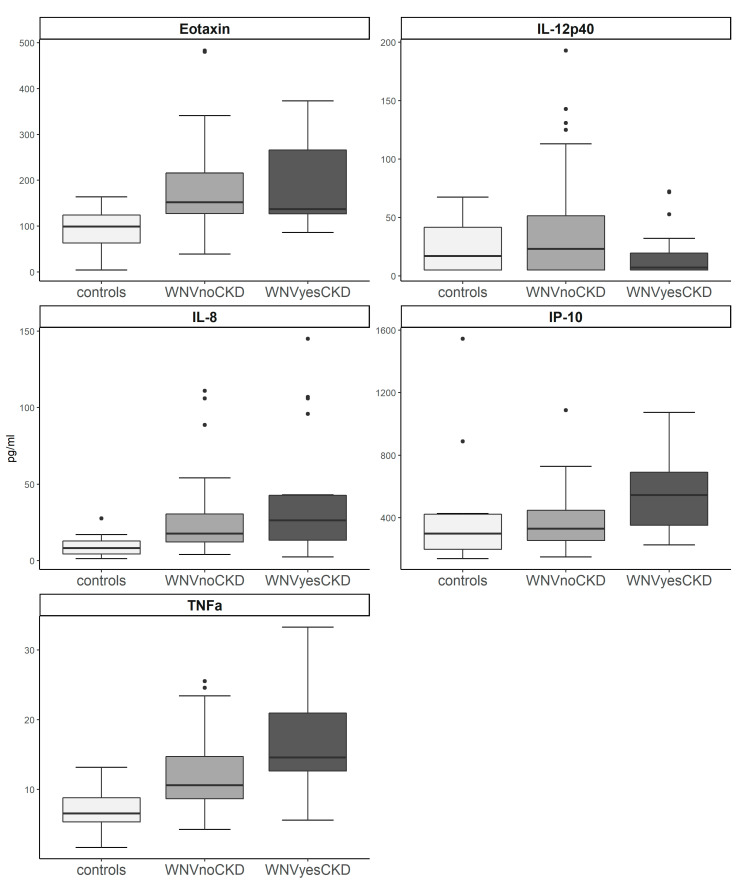
Cytokine values per WNV exposure group. Serum samples were drawn from study participants at last time points of data collection and were analyzed using a Luminex^®^ IS 100 platform (Austin, TX, USA) to detect 26 different cytokines. Of all 26 cytokines, five were found to have a statistically meaningful difference between two of the three possible groups (statistical significance was reduced to 0.0019 due to multiple comparisons). Eotaxin and IL-12p40 were both found to have meaningful differences between the controls and those with history of WNV without CKD (*p*-value = 0.0013 and 0.0002, respectively). IL-8 and TNFα were both found to have meaningful differences between controls and individuals with history of WNV and subsequent development of CKD (*p*-value < 0.0001, both). Lastly, IP-10 was found to be significantly higher in individuals with CKD and history of WNV infection compared to those with history of WNV and no evidence of CKD (*p*-value = 0.0017).

Cytokine analyses measured concentrations as picograms per milliliter (pg/mL) and were performed on samples from all participating individuals in the WNV-positive group (*n* = 89) and a randomly selected subset of controls (*n* = 12). WNV participants were further subdivided into those who did not develop CKD (*n* = 68) and those who developed CKD (*n* = 21). A post-hoc power calculation using PASS software (NCSS LLC., Kaysville, UT) showed that our study’s type-II error probability was summarized with a Beta value greater than 0.95. After adjusting our *p*-value significance level for 26 multiple comparisons, we found that eotaxin, IL8, IL-12p70, IP-10, and TNFα all had a *p*-value less than 0.0019, as required by the Bonferroni correction for significance (Table 3). Further analysis using Dunn’s test revealed that eotaxin and IL-12p70 were distinctly elevated in WNV-positive participants without CKD when compared to healthy controls (*p*-values 0.0013 and 0.0002, respectively). Meanwhile, IL-8 and TNFα were elevated in WNV-positive participants with CKD when compared to healthy controls (*p*-values 0.0001 and <0.0001, respectively), and IP-10 was elevated in WNV participants with CKD as compared to those without CKD (*p*-value 0.0017) (Table 4). However, the trend in all five of the markers consistently showed that the WNV with CKD group always had higher values than the WNV non-CKD counterpart, although the differences were not always statistically significant (Figure 1). This trend was found in several other cytokines that had statistical *p*-values < 0.05, but did not meet criteria for the corrected *p*-value of 0.0019 (i.e., IL-1α, MIP1α, and MIP1β), with one exception being IL-4, which had a lower average in the WNV with CKD group than those without CKD.

## 4. Discussion

This WNV patient cohort provided an opportunity to improve our understanding of the long-term impact of the disease process. The prevalence of CKD in WNV-positive cases (23.6%) was greater than the national average of 4–15% in the United States for individuals with similar ages to our population [32]. By following these individuals over time and confirming the diagnoses of WNV and CKD, this study further supports the correlation between these two diseases. After accounting for potential confounding, we were able to confirm that WNV remains an independent risk factor, increasing the risk for later development of CKD by a factor of 1.9.

These results support the work previously published by Nolan et al., while further exploring potential cytological factors associated with the correlation. As expected, hypertension and age were associated with the development of CKD. However, there were no independent associations found with diabetes mellitus, obesity, or heart disease after adjustment. This remains consistent with our previously published cross-sectional analysis in the WNV cohort population that found CKD to be significantly associated with age and hypertension, while diabetes mellitus only had a mild to negligible effect [7]. While we know that diabetes plays a role in kidney disease, it is likely that our sample size was simply not large enough to detect significance after adjustment. 

Additionally, our cytokine analysis not only confirmed that WNV-infected individuals sustained elevated levels of inflammatory markers years after infection [4], but those who developed CKD had even higher levels of circulating cytokines. This suggests these individuals continue to undergo an inflammatory process or potentially active viral disease, either of which may explain the chronic complications related to the kidneys and brain. After applying the Bonferroni correction, eotaxin, IL-8, IL-12p70, IP-10, and TNFα were distinctly elevated in individuals with a history WNV infection. Amongst WNV patients, only IP-10 was specifically elevated in WNV patients with CKD as compared to WNV patients without CKD. These cytokines have been identified in similar WNV studies looking at acute and chronic complications of the disease [16,33,34]. Acute infections have demonstrated robust expression of IL-1B, IL-6, IL-8, MIP1a, and TNFα as the key mediators driving immediate cell death. This is often followed by a rapid and sustained rise in IP-10 that, in combination with IL-1B and TNFα, is largely responsible for monocyte recruitment [6,16]. This process has been well studied in an attempt to understand how the virus introduces itself through the blood–brain barrier. At a systems and genetics level, IP-10 has repeatedly been demonstrated to be associated with viral load and disease severity in WNV cases [18,35]—a pattern that is consistent with other viruses, including hepatitis C virus, dengue virus, and hantavirus [36,37,38]. 

Complications resultant of WNV infection occur with little elaboration on the immune physiology [39,40]. When comparing WNV patients with and without symptoms, significant elevation of IL-12p70, IL-2, IL-6, IL-8, and IP-10 was observed. More specific analyses using differential gene expression studies between asymptomatic and severe WNV subjects found that IL-8 and TNF families were being significantly expressed after adjustment for age up to ten years after viral exposure [18,41]. The recurrence of these pro-inflammatory markers in our population reflects the known disease pathology of WNV and shows no resemblance to the cytokine profiles of any chronic or non-infectious cause of CKD [23]. Conversely, in WNV and other infections, elevated levels of IL-4 appear to be associated with less-severe forms of the disease [42]. Even years after infection, WNV patients that had mild or asymptomatic phenotypes at the time of infection will continue to have higher levels of IL-4 as compared to those with more severe symptoms, suggesting that IL-4 is possibly protective [41]. In our study, we found a similar relationship: IL-4 was the only cytokine in the CKD group that, on average, was less than their WNV non-CKD counterparts. While nearly every cytokine was found to be greater in the WNV patients compared to controls, the greater granularity we can provide will better describe the pathological signature of WNV physiology. This reiterates what we already know about WNV; it remains difficult to distinguish unique characteristics of the WNV group with CKD. Overall, many of the cytokines that were found to be positively associated with the CKD group are generally associated with inflammation. IP-10 is a notable exception, and was the only marker that was statistically different in the WNV with CKD group compared to WNV individuals without CKD. This suggests a more active viral disease process taking place, likely in the kidneys, rather than simply a prolonged inflammatory state. Evidence of viral replication in renal tissue has been identified before [9], but more research is necessary.

It is important to note that age was the most difficult confounding variable to accurately adjust for, which limits the generalizability of our results. We found that in both WNV-positive and control participants, CKD was only diagnosed in individuals that were at least 50 years of age; therefore, we can only accurately interpret our findings to that age group. We also note that the interpretation of cytokine characterization in older individuals is subject to the effects of immunosenescence, the increasingly recognized phenomenon that our immune response changes as we age [43]. We were only able to find a significant correlation between age and cytokine levels in 3 of the 26 markers that were measured (IL-5, IL-7, and TNFα), but our attempts to account for age with greater specificity in our model were limited due to our sample size, leaving us unable to converge the model beyond dichotomization above and below the age of 65. Our cytokine analysis additionally had challenges associated with the persistence of outliers with higher values and a truncation effect at the lower values due to the lower limit of quantitation of the cytokine test. These barriers factored into some inconsistencies with our results; for example, the greater average serum IP-10 concentration in the control group as compared to WNV individuals without CKD was found to be due to one or two specific outliers. A global evaluation of the raw cytokine values in the control group showed that there were two individuals that had markedly elevated cytokine levels that would not be anticipated for a healthy control. Despite efforts to account for these outliers, their impact still lingers in our dataset. In fact, the prevalence of comorbidities in our control population was higher than expected, likely due to our recruitment of controls from a family medicine clinic where people with underlying health conditions might frequent more regularly than true healthy controls. While our analysis technique appropriately accounts for this non-normal distribution, it does highlight the challenge of using and interpreting serum cytokines that can be elevated for a multitude of reasons beyond those questioned in this study. Overall, these issues add noise to the data, but do not take away the significance of the results, and suggest that further discrimination between each group may be hidden.

In conclusion, this study supports the importance of CKD in the context of WNV infection. Additional research in this area is necessary, with a greater focus on distinguishing differences between WNV patients with and without CKD. Exploring the genetic and histological differences between WNV cases with and without CKD will also be necessary to fully understand this disease’s process and improve patient outcomes.

## Figures and Tables

**Table 1:** Patient demographics.

Patient Demographics	WNV-Negative Controls *n* = 127	WNV-Positive Cases *n* = 89	*p*-Value
Sex			
Male, *n* (%)	68 (53.5%)	54 (61.3%)	0.37
Age, mean (std. dev.)	55.61 (14.8)	58.84 (16.4%)	0.13
Race, *n* (%)			
Caucasian	116 (91.3%)	81 (91.0%)	0.99
African American	8 (6.3%)	6 (6.8%)	0.99
Asian/Pacific Islander	3 (2.4%)	1 (1.1%)	0.88
Ethnicity, *n* (%)			
Hispanic	12 (9.5%)	3 (3.4%)	0.15
CKD Risk Factors, *n* (%)			
Obesity	46 (36.2%)	22 (31.9%)	0.10
Diabetes Mellitus	23 (18.1%)	16 (18.0%)	0.99
Hypertension	61 (48.0%)	33 (37.1%)	0.14
Heart Disease	5 (3.9%)	13 (14.6%)	0.01
Renal Involvement, *n* (%)			
CKD *	16 (12.6%)	21 (23.6%)	0.13

* Chronic kidney disease (CKD) was defined as a calculated eGFR < 60 mL/min lasting 3 months or more.

**Table 2:** Log binomial regression analysis adjusted for meaningful CKD risk factors among both WNV-positive cases and WNV-negative controls.

Risk Factor	Adjusted Risk Ratio (95% CI)
Hypertension	3.13 (1.26–7.77) *
Age ≥ 65	2.46 (1.18–5.09) *
History of WNV	1.91 (1.13–3.25) *
Diabetes mellitus	1.63 (0.89–3.04)
Obesity (BMI > 30)	1.43 (0.71–2.87)
History of heart disease	0.78 (0.43–1.41)

* Indicates statistical significance (*p*-value < 0.05). Abbreviations: WNV, West Nile virus; BMI, body mass index.

**Table 3:** Mean Concentration (pg/mL) and Standard Deviation (std. dev.) of 26 Cytokines in Serum with Comparison between the Three Groups.

Cytokine/Chemokine	WNV-Negative Controls (*n* = 12) Mean (std. dev.)	WNV No CKD (*n* = 68) Mean (std. dev.)	WNV w/ CKD (*n* = 21) Mean (std. dev.)	Kruskal–Wallis *H*-Test *p*-Value
Eotaxin	93.42 (47.55)	173.78 (83.35)	189.96 (82.84)	***p* = 0.0011**
G-CSF	27.20 (20.64)	41.34 (37.88)	32.17 (20.07)	*p* = 0.5148
GM-CSF	3.31 (2.07)	7.68 (11.99)	6.25 (7.35)	*p* = 0.2438
INF-α2	14.45 (12.23)	24.20 (24.40)	18.08 (15.36)	*p* = 0.4642
INF-γ	4.47 (4.07)	17.21 (29.59)	8.02 (7.14)	*p* = 0.4483
IL1α	6.42 (12.21)	30.45 (48.58)	40.50 (85.01)	*p* = 0.0369
IL1β	5.16 (4.01)	7.52 (11.33)	6.10 (16.43)	*p* = 0.2930
IL2	1.65 (0.12)	2.30 (1.70)	2.82 (3.85)	*p* = 0.2890
IL3	1.65 (0.28)	1.68 (0.50)	1.56 (0.00)	*p* = 0.2228
IL4	9.53 (8.96)	34.23 (30.88)	32.31 (29.37)	*p* = 0.0054
IL5	1.98 (1.24)	1.78 (1.25)	1.38 (0.65)	*p* = 0.2867
IL6	2.24 (1.37)	5.66 (5.52)	6.73 (7.77)	*p* = 0.0891
IL7	6.05 (3.94)	7.42 (6.16)	4.58 (3.41)	*p* = 0.1693
IL8	9.68 (7.61)	25.44 (21.40)	40.76 (41.16)	***p* = 0.0014**
IL10	6.26 (5.23)	6.77 (5.57)	7.37 (5.42)	*p* = 0.6938
IL12p40	25.80 (24.05)	37.41 (41.20)	18.41 (22.53)	*p* = 0.1230
IL12p70	3.02 (0.39)	12.76 (21.50)	13.65 (25.52)	***p* = 0.0003**
IL13	4.99 (6.49)	6.49 (10.46)	2.45 (2.77)	*p* = 0.2990
IL15	3.83 (2.11)	3.76 (2.14)	3.00 (1.07)	*p* = 0.5175
IL17α	3.23 (3.63)	8.8 (18.41)	19.04 (65.53)	*p* = 0.0834
IP-10	447.45 (420.73)	363.15 (158.05)	556.67 (241.53)	***p* = 0.0018**
MCP1	398.58 (207.15)	534.87 (194.63)	563.20 (205.88)	*p* = 0.0749
MIP1α	3.05 (0.96)	11.73 (12.02)	12.77 (13.43)	*p* = 0.0242
MIP1β	38.80 (27.90)	60.43 (33.73)	86.62 (43.96)	*p* = 0.0028
TNFα	7.29 (3.71)	11.91 (4.76)	16.44 (6.70)	***p* < 0.0001**
TNFβ	6.99 (5.71)	11.29 (14.84)	5.38 (4.46)	*p* = 0.1351

Results of analyses were adjusted for multiple inter-group comparisons using the Bonferroni method. Abbreviations: WNV, West Nile virus; CKD, chronic kidney disease; G-CSF, granulocyte-colony stimulating factor; GM-CSF, granulocyte-macrophage colony stimulating factor; INF, interferon; IL, interleukin; IP-10, interferon gamma-induced protein 10; MCP1, monocyte chemoattractant protein-1; MIP, macrophage inflammatory protein; TNF, tumor necrosis factor.

**Table 4:** Dunn’s test for inter-group post hoc comparison of cytokine profiles.

Cytokine	Healthy Controls vs. WNV no CKD	Healthy Controls vs. WNV w/CKD	WNV no CKD vs. WNV w/CKD
Eotaxin	93.42 (47.55) vs. 173.78 (83.35) ***p* = 0.0013**	93.42 (47.55) vs. 189.96 (82.84) *p* = 0.0026	173.78 (83.35) vs. 189.96 (82.84) *p* = 0.9999
IL-8	9.68 (7.61) vs. 25.44 (21.40) *p* = 0.0035	9.68 (7.61) vs. 40.76 (41.16) ***p* = 0.0001**	25.44 (21.40) vs. 40.76 (41.16) *p* = 0.9347
IL-12p70	3.02 (0.39) vs. 12.76 (21.50) ***p* = 0.0002**	3.02 (0.39) vs. 13.65 (25.52) *p* = 0.0068	12.76 (21.50) vs. 13.65 (25.52) *p* = 0.9999
IP-10	447.45 (420.73) vs. 363.15 (158.05) *p* = 0.9999	447.45 (420.73) vs. 556.67 (241.53) *p* = 0.0416	363.15 (158.05) vs. 556.67 (241.53) ***p* = 0.0017**
TNFα	7.29 (3.71) vs. 11.91 (4.76) *p* = 0.0112	7.29 (3.71) vs. 16.44 (6.70) ***p* < 0.0001**	11.91 (4.76) vs. 16.44 (6.70) *p* = 0.0169

Results of analyses were adjusted for multiple inter-group comparisons using the Bonferroni correction. Abbreviations: WNV, West Nile virus; CKD, chronic kidney disease; IL, interleukin; IP-10, interferon gamma-induced protein 10; TNF, tumor necrosis factor.

## Data Availability

The data presented in this study are available on request from the corresponding author.

## Supplementary Materials

The following are available online at https://www.mdpi.com/1999-4915/13/2/311/s1.
