# Supplementary material for: Unique Cytokine Response in West Nile Virus Patients Who Developed Chronic Kidney Disease: A Prospective Cohort Study
Source: Viruses. 2021 Feb 17;13(2):311. doi: 10.3390/v13020311 (PMC7922428; doi:10.3390/v13020311)
Supplement: Supplementary file 1 [file viruses-13-00311-s001.pdf]

Supplemental Table S1. Cytokines Associated with Known Arboviral Diseases and Organ Failure

Cytokine	Known Association with Arboviral Disease	Known Association with Infection Related Organ Damage
Eotaxin	Chikungunya virus	
G-CSF	Chikungunya virus	
GM-CSF	Chikungunya virus, Dengue virus, Hantavirus, Yellow Fever virus, Zika virus	
IFN-α2	Chikungunya virus, West Nile virus	
IFN-γ	Chikungunya virus, Dengue virus, West Nile virus, Yellow Fever virus, Zika virus	Renal Injury
IL1α	Hantavirus, Yellow Fever virus	
IL1β	Hantavirus, West Nile virus, Yellow Fever virus, Zika virus	Renal Injury, Hepatic Injury, Neurological Injury
IL2	Zika virus	Renal Injury
IL3		
IL4	Chikungunya virus, Yellow Fever virus, Zika virus	
IL5		
IL6	Chikungunya virus, BK virus, Dengue virus, Hantavirus, West Nile virus, Zika virus	Renal Injury, Hepatic Injury, Neurological Injury
IL7		
IL8	Chikungunya virus, Dengue virus, Hantavirus, West Nile virus, Yellow Fever virus, Zika virus	Hepatic Injury
IL10	Dengue virus, Yellow Fever virus, Zika virus	Renal Injury, Multi-Organ Dysfunction
IL12p40		Multi-Organ Dysfunction
IL12p70		
IL13	Zika virus	Renal Injury
IL15	Dengue virus	
IL17α	Chikungunya virus, Zika virus	Renal Injury
IP-10	Chikungunya virus, Hantavirus, West Nile virus, Zika virus	Multi-Organ Dysfunction
MCP1	Chikungunya virus, Dengue virus, West Nile virus	Renal Injury
MIL1α		
MIL1β	Dengue virus	
TNFα	Dengue virus, Hantavirus, West Nile virus, Yellow Fever virus, Zika virus	Renal Injury, Hepatic Injury, Neurological Injury
TNFβ	Hantavirus, West Nile virus	

Elevated cytokine levels were detected in acute, recovery, and chronic phases.

Abbreviations: G-CSF, granulocyte-colony stimulating factor; GM-CSF, granulocyte-macrophage colony stimulating factor; INF, interferon; IL, interleukin; IP-10, interferon gamma-induced protein 10; MCP1, monocyte chemoattractant protein-1; MIP, macrophage inflammatory protein; TNF, tumor necrosis factor.
